# Comorbidity in bullous pemphigoid: up-date and clinical implications

**DOI:** 10.3389/fimmu.2023.1196999

**Published:** 2023-06-29

**Authors:** Johanna Huttelmaier, Sandrine Benoit, Matthias Goebeler

**Affiliations:** Department of Dermatology, Venereology and Allergology, University Hospital Würzburg, Würzburg, Germany

**Keywords:** bullous pemphigoid, autoimmune skin blistering disease, comorbidity, neurologic disease, metabolic disease, malignancy, inflammatory disease

## Abstract

Bullous pemphigoid is the most common autoimmune blistering disease in industrialized countries and particularly affects the elderly. In this patient population, comorbid diseases are frequent and may complicate management and treatment of bullous pemphigoid. A better understanding why distinct diseases are more frequent in bullous pemphigoid patients may lead to new pathophysiological insights and - as a consequence - result in better patient care. The association of bullous pemphigoid with neurological and psychiatric diseases is well known and confirmed by several case-control studies. Association with further diseases such as malignancy and metabolic diseases are still discussed controversially. In recent years new relationships between bullous pemphigoid and autoimmune as well as inflammatory skin diseases have been reported. This review provides a systematic overview on studies addressing comorbidity in bullous pemphigoid patients. Increasing the awareness of both, common and rare comorbid diseases, may enable clinicians to optimize patient support and individualized treatment of bullous pemphigoid.

## Introduction

Bullous pemphigoid (BP) is the most common autoimmune blistering disease (AIBD) with an incidence of approximately 20 cases per million people in Germany (1). In recent years the incidence has been rising, presumably due to an ageing population and increasing prescription of certain drugs (2).

BP patients typically present with urticarial plaques, vesicles, blisters and erosions and often suffer from intense pruritus (1). In approximately 20% of patients itching is the main symptom, often being associated with non-bullous prurigo-like nodules. In severe cases the mucous membranes may be affected as well. BP is characterized by autoantibodies directed against structural proteins of the dermal–epidermal junction, especially BP-180. The latter connects keratinocytes to the basement membrane. Binding of specific autoantibodies leads to inflammation with subsequent disruption of the dermal-epidermal junction and blister formation (3).

The mean age of onset of BP is 78 years (1). As the incidence of BP is increasing with age patients often suffer from a variety of comorbid diseases. Comorbidity is defined as the coexistence of different diseases in one individual with an index disease, e.g. BP. Importantly, comorbidity is not a direct result or complication of the index disease itself but is defined by multiple diseases existing simultaneously in one individual regardless of their causal relationship (4). Comorbidity and resulting polypharmacy may delay and complicate treatment of BP, thus awareness of both common and rare comorbid diseases of BP may enable clinicians to optimize patient support and treatment.

In 2019 a 1-year combined mortality rate in patients with BP of 26.7% in Europe and 15.1% in the United States was reported (5). Recently, a retrospective cohort study including 148 Romanian patients with BP showed similar results with overall survival rates of 74.2% after one year, 53.4% after three years, 43.6% after five years and 31.3% after 10 years (6). Among others, advanced age, neurological disease, valvular heart disease and malignancies were associated with higher mortality rates, however, results were not validated by comparison to a control group. A retrospective analysis of patients with BP in Korea showed a 1.83-fold increased standardized mortality ratio as compared to age- and sex-matched controls (7). The authors identified age over 70 years, cardiac and renal disease as risk factors for increased mortality. However, other associated diseases such as hypertension, stroke, neurological disease, pulmonary disease, dementia and malignant diseases showed no significant differences in mortality rates.

Most studies assessing comorbid diseases in BP focus on a single disease or a group of diseases; reports on the overall prevalence of comorbidities are sparse. A Spanish retrospective observational study including 5,424 patients with BP reported an age-adjusted Charlson Comorbidity Index with significantly higher scores in the pemphigoid compared to the pemphigus group (6.0 vs. 4.7, p < 0.001) indicating a higher prevalence for comorbidity in these patients (2). In line, increased prevalence of arterial hypertension, kidney disease, diabetes, heart failure, dementia, chronic obstructive pulmonary disease and Parkinson’s disease was observed.

The association of BP with neurological and psychiatric diseases is well known and has been confirmed by several case-control or cohort studies (8–13). Association with malignancy and metabolic diseases is still discussed controversially (13–18). In recent years new relationships between BP and autoimmune as well as inflammatory skin diseases have been reported (19–21).

Given the heterogeneity of studied patient cohorts and variable study designs, comparison and interpretation of published observations is quite challenging. Nevertheless, we herein provide a comprehensive overview of comorbid diseases in BP patients.

## Methods

### Searching strategy and selection criteria

Literature search was performed using the PubMed database for reports published between January 1990 and November 2022. Searches were limited to publications in English and German language. Medical subject heading terms used in the literature search included ‘‘bullous pemphigoid’’ AND “comorbidities”, ‘‘bullous pemphigoid’’ AND “comorbidity”, “subepidermal autoimmune blistering” AND “comorbidities” and “subepidermal autoimmune blistering” AND “comorbidity”. Additionally, 11 studies were identified *via* citation in other publications.

## Results

**Figure 1:**
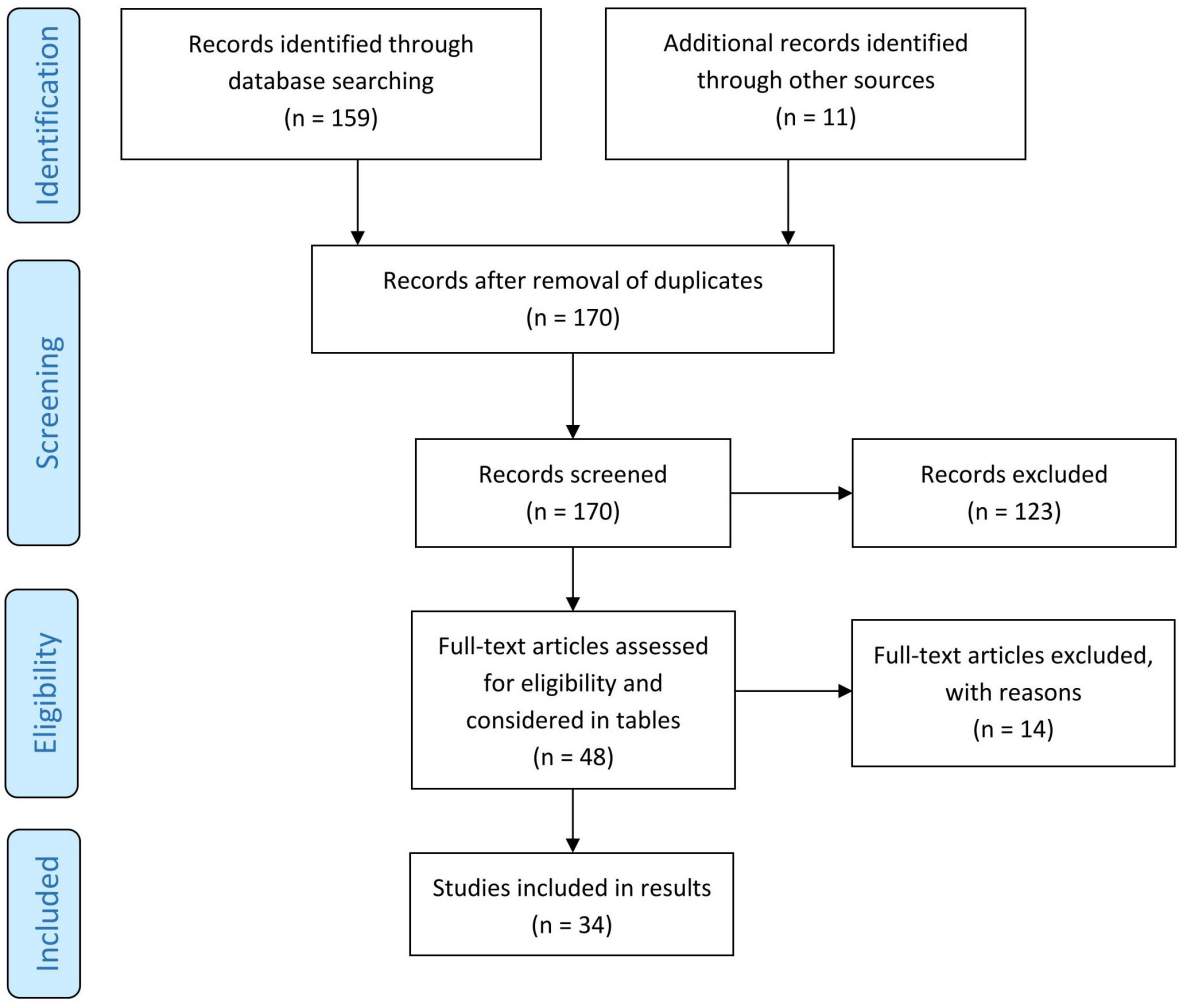
Screening method for published studies. [adapted according to the PRISMA statement (22)].

Altogether, our search strategy revealed 170 articles dealing with BP and comorbid diseases (**Figure 1**). Of those, 48 were original articles with 23 case-control, 21 cohort or register studies, two cross-sectional studies and two meta-analyses of cross-sectional, case-control and cohort studies.

### Neurologic and psychiatric diseases

Of the considered 48 studies 17 studies discussed neurologic and psychiatric comorbidities in patients with BP (**Table 1**). The association of BP with neurologic and psychiatric diseases is well acknowledged and confirmed by several case-control and cohort studies (8–13). In 26.4 – 55.8% of patients with BP at least one neurological disease was documented compared to 9.1 – 20.5% in controls depending on the study criteria and designs (9, 24, 28). We recently published a case-control study including 300 patients and 598 age- and sex-matched controls evaluating neurologic and psychiatric comorbidities in BP with a documented neurologic disease in 49.0% and psychiatric disease in 20.3% of BP patients compared to 23.0% and 9.3% in controls, respectively (13). A Taiwanese population-based study identified stroke (36.8%), dementia (17.7%), Parkinson’s disease (11.9%) and epilepsy (5.8%) as the most commonly associated neurologic diseases (20). A 3.63-fold increased risk for any neurological disease preceding the diagnosis of BP was calculated. Interestingly, multiple sclerosis (MS) was not diagnosed in any of the 3,485 evaluated patients. However, various other cohort and case-control studies identified MS to be strongly associated with BP (10, 13, 27). A study analyzing the Finnish Care Register identified MS to be the strongest risk factor for developing BP [Odds ratio (OR) 5.9; 95% CI 3.9-8.5] (8). A Danish register-based cohort study including 3,281 patients with BP described an increased prevalence of MS at time of diagnosis (OR 9.7; 95% CI 6.0–15.6) (10). Noteworthy, during the follow-up period the hazard ratio (HR) for development of MS was significantly higher in patients with BP than in controls (HR 9.4; 95% CI 4.9–18.0).

**Table 1:** Association between bullous pemphigoid and neurologic and psychiatric diseases.

Author	Year	Location	Study design	Reference cohort	N (case/control)	Neurologic/psychiatric comorbidities in individuals with BP	OR/*HR*	95% CI	*p*
Chen Y.J., et al. (20)	2011	Taiwan	Case-control	Age- and sex-matched controls	3,485/17,425	**Dementia** **Epilepsy** **Parkinson’s disease** **Stroke** **Schizophrenia**	**4.81** **3.97** **3.49** **3.30** **2.56**	**4.26–5.42** **3.28-4.81** **3.05-3.98** **3.03-3.60** **1.56-4.30**	**< 0,003** **< 0,003** **< 0,003** **< 0,003** **< 0,003**
Yang Y.W., et al. (23)	2011	Taiwan	Cohort-study	Age- and sex-matched controls	390/1,950	**Stroke**	**2.37**	**1.78-3.15**	**<0.001**
Teixeira V. B., et al. (24)	2014	Portugal	Case-control	Age- and sex-matched controls	77/178	**Stroke** **Dementia**	**5.22 4.13**	**1.65-15.51** **1.42-14.5**	**<0.001** **<0.001**
Kwan Z., et al. (25)	2015	Malaysia	Case-control	Age-, sex- and ethnicity-matched controls	43/43	**Neurological disorder** **Dementia** Stroke Parkinson’s disease Epilepsy	**3.5** **n.a.** 2.1 n.a. n.a.	**1.2-10.2** **n.a.** 0.6-7.2 n.a. n.a.	**0.026** **0.002** 0.237 1.0 0.494
Försti A.K., et al. (8)	2016	Finnland	Cohort study	Age- and sex-matched controls	4,524/66,138	**Schizophrenia** **Personality disorders**	**2.7** **2.2**	**2.0-3.5** **1.3-3.3**	**<0.05** **<0.05**
Sim B., et al. (26)	2017	Singapore	Case-control	Age- and sex-matched controls	105/315	**Parkinson’s disease** **Stroke** **Dementia** **Epilepsy** **Depression** Schizophrenia	**20.59** **6.24** **5.89** **3.75** **3.73** 4.37	**4.69-90.5** **3.48-11.2** **2.87-12.0** **1.01-13.9** **1.49-9.37** 0.37-51.3	**<0.001** **<0.001** **<0.001** **0.049** **0.005** 0.24
Daneshpazhooh M., et al. (9)	2017	Iran	Case-control	Age- and sex-matched controls	160/317	**Stroke** **Any neurological disease** **Dementia** Epilepsy Parkinson’s disease Multiple sclerosis	**4.96** **3.53** **3.09** 4.04 1.14 1.00	**2.49–9.88** **2.1–5.90** **1.08–8.84** 0.73-22.3 0.33-3.94 0.98-1.01	**< 0.001** **< 0.001** **< 0.03** 0,08 0,84 0,5
Kibsgaard L., et al. (10)	2017	Denmark	Matched Cohort study	n.a.	3,281/32,213	**Multiple sclerosis** **Parkinson’s disease** **Stroke** **Alzheimer’s disease**	***9.7*** ***4.2*** ***2.7*** ***2.6***	**6.0 – 15.6** **3.1 – 5.8** **2.4 – 2.9** **1.8 – 3.5**	**<0.05** **<0.05** **<0.05** **<0.05**
Ren Z., et al. (27)	2017	USA	Register study of hospitalized adult patients in the USA	n.a.	2,108 BP primary diagnosis 11,234 BP secondary diagnosis 72,108,077 total hospital discharges	**Multiple sclerosis** **Demyelinating disorders** **Presenile dementia** **Paralysis** **Other neurological disorders** **Parkinson’s disease** **Other dementias** **Vascular dementia** **Senile dementia** **Epilepsy** **Psychoses** **Depression**	**4.07** **3.57** **2.63** **2.52** **1.98** **1.86** **1.71** **1.67** **1.67** **1.67** **1.25** **1.19**	**3.22-5.14** **1.48-8.61** **1.34-5.18** **2.30-2.77** **1.65-2.10** **1.86-2.11** **1.51-1.94** **1.40-2.00** **1.41-1.98** **1.41-1.98** **1.12-1.39** **1.11-1.28**	**0.0003** **0.01** **0.01** **0.0003** **0.0003** **0.0003** **0.0003** **0.0003** **0.0003** **0.0003** **0.0003** **0.0003**
Kalińska-Bienias A., et al. (28)	2019	Poland	Case-control	Age- and sex-matched controls	218/168	**Dementia** **Neurologic disease**	**7.89** **3.76**	**2.99-20.85** **2.13-6.65**	**<0.001** **<0.001**
Papakonstantinou E., et al. (29)	2019	Germany	Cohort-study	Age- and sex-matched controls	183/348	**Neurological disorder** **Dementia** **Stroke** **Parkinson’s disease** Alzheimer’s disease	**10.8** **9.9** **4.1** **2.9** 0.3	**6.9–16.7** **5.4-17.8** **1.7-9.7** **1.0-8.4** 0.0-7.9	**<0.001** **<0.001** **0.0015** **0.0434** 0.5303
Rania M, et al. (12)	2020	Denmark	Cohort study (national register)	Adjustment for medication	2,892/6,470,450	Subsequent BP in **- Any psychiatric disorder** **- Organic disorder** **- Substance use disorder** **- Schizophrenia** **- neurotic disorder** **- personality disorder** **- intellectual disorder**	***1.4*** ***1.55*** ***1.70*** ***1.57*** ***1.41*** ***1.53*** ***2.96***	**1.25-1.56** **1.32-1.81** **1.35-2.13** **1.21-2.02** **1.16-1.71** **1.22-1.92** **1.58-5.51**	**n.a.** **n.a.** **n.a.** **n.a.** **n.a.** **n.a.** **n.a.**
Kridin K., et al. (11)	2021	Germany	Cross-sectional study	Age- and sex-matched controls	1,743/10,141	**Parkinson’s disease** **Epilepsy** **Alzheimer’s disease** **Stroke**	**2.71** **2.18** **2.11** **1.84**	**2.19-3.35** **1.72-2.77** **1.73-2.57** **1.55-2.19**	**<0.001** **<0.001** **<0.001** **<0.001**
Kilic Sayar S., et al. (30)	2021	Turkey	Case-control	Age- and sex-matched controls	145/310	**Stroke** **Neurological disorder** Parkinson’s disease Alzheimer’s disease Dementia	**2.29** **1.94** 1.28 1.69 2.20	**1.15-4.56** **1.12-3.35** 0.30-5.46 0.61-4.64 0.81-5.99	**0.017** **0.017** 0.731 0.304 0.122
Sánchez-García V., et al. (2)	2022	Spain	Retrospective observational study	Compared to pemphigus patients	5,424/1,950	Higher prevalence in BP compared to pemphigus **- Dementia** **- Parkinson’s disease** - Cerebrovascular disease - Depression - Epilepsy - Multiple sclerosis	**n.a.** **n.a.** n.a. n.a. n.a. n.a.	**n.a.** **n.a.** n.a. n.a. n.a. n.a.	**<0.001** **0.043** 0.057 0.087 0.09 0.074
Martin E., et al. (13)	2022	Germany	Case-control	Age- and sex-matched controls	300/583	**Multiple sclerosis** **Dementia** **Organic brain syndrome** **Neurological disease** **Parkinson’s disease** **Psychiatric disease** **Epilepsy** **Stroke** **Depression**	**10.00** **7.47** **5.50** **3.59** **3.03** **2.441** **2.397** **2.355** **1.799**	**1.16–85.59** **4.45–12.54** **1.75–17.27** **2.56–5.02** **1.51–6.08** **1.64-3.61** **1.11–5.14** **1.60–3.45** **1.15–2.80**	**<0.05** **<0.0005** **<0.05** **<0.0005** **<0.05** **<0.0005** **<0.05** **<0.0005** **<0.05**
Titou H., et al. (31)	2022	Morocco	Case-control	Age- and sex-matched controls	81/162	**Significant association of BP with any neurological disease and stroke**	**n.a.**	**n.a.**	**0.001**

Data in bold indicate statistically significant results.

BP, bullous pemphigoid; OR, odds ratio; HR, hazard ratio; CI, confidence interval; n.a, not available.

Dementia is also commonly reported in patients with BP. Nevertheless, studies are inconsistent and partially incomparable as the subtypes of dementia are often not differentiated and reported. Analysis of hospitalized patients with BP in the United States of America showed an association of BP with numerous forms of dementia such as presenile dementia (OR 2.63; 95% CI 1.34-5.18), vascular dementia (OR 1.67; 95% CI 1.40–2.00), senile dementia (OR 1.67; 95% CI 1.41–1.98) and other dementias (OR 1.71; 95% CI 1.51–1.94) (27). A German retrospective population-based study comprising 1,743 patients with BP who were matched with 10,141 controls showed a significant association with Alzheimer’s disease (OR 2.11; 95% CI 1.73–2.57; P < 0.001) (11). The same study identified a significantly increased life-time prevalence for Parkinson’s disease in patients with BP compared to age- and sex-matched controls (OR 2.71; 95% CI 2.19–3.35; p < 0.001). This observation is consistent with studies from Taiwan and the United States that showed an OR of 3.49 and 1.86, respectively. Interestingly, a case-control study from Portugal including 77 patients with BP identified an OR of 4.91 for Parkinson’s disease; however, after multivariate analysis the association with Parkinson’s disease did not prove to be statistically significant (24). This was possibly due to the small number of patients.

The above mentioned German retrospective population-based study including 1,743 patients with BP identified an increased life-time prevalence for stroke (OR 1.84; 95% CI 1.55–2.19; p < 0.001) (11). In line with these results, a population-based follow-up study from Taiwan including 390 patients and 1,950 matched controls observed 89 events of stroke (22.8%) in patients with BP compared to 223 events (11.4%) in controls during a 3-year follow-up period (p < 0.001) (23). The calculated HR for stroke in patients with BP was 2.37 (95% CI 1.78-3.15; p < 0.001) after multivariate adjustment for confounders such as hypertension, diabetes, hyperlipidemia, heart failure, atrial fibrillation and coronary heart disease.

Not only neurological but also psychiatric disorders seem to be associated with BP as mentioned above. A Danish register-based cohort study focusing on psychiatric disorders identified a significantly elevated risk for psychiatric disorders (HR 1.37; 95% CI 1.15–1.63) and specifically for organic disorders (ICD-10 Code F0) (HR 1.27; 95% CI 1.04–1.54) after adjusting for medication (12). In patients with a diagnosed psychiatric disorder, the risk of development of BP was increased (HR 1.79; 95% CI 1.61–1.99) with a mean latency of 14.6 years. Subgroup analysis identified significantly higher risks for individuals with prior intellectual disorder (ICD-10 code F7) (4.18-fold), substance abuse (ICD-10 code F1) (2.32-fold), schizophrenia (ICD-10 code F2) (2.01-fold), personality disorders (ICD-10 code F60) (2.01-fold), organic disorders (ICD-10 code F0) (1.92-fold) and neurotic disorders (ICD-10 code F4) (1.85-fold). In contrast, a recently published meta-analysis of cohort studies calculated that patients with BP exhibited no significantly increased risk for depression (HR 1.09; 95% CI 0.94-1.26) and schizophrenia (HR 1.35; 95% CI 0.76-2.39) (32). The authors hypothesized that these contradictory results are possibly due to an induction of psychiatric disorders like depression by steroid treatment of BP. Another Taiwanese case-control study reported that the association of BP with schizophrenia occurred predominantly in female patients, which may also explain these inconsistencies (20).

### Metabolic diseases

Eighteen of the selected 48 studies discussed metabolic diseases in patients with BP (**Table 2**). Apart from neurologic disorders metabolic diseases such as diabetes mellitus, arterial hypertension, cardiac diseases and renal impairment appear to be associated with BP (7, 40, 42, 43). Individuals suffering from BP showed a significantly higher prevalence of metabolic syndrome than matched controls (35.2% vs. 14.8%; p < 0.001) (43). BP patients with metabolic syndrome were older at time of diagnosis than BP patients without metabolic syndrome (72.0 vs. 60.0 years, p = 0.006). Overall, patients with BP have a higher risk for cardiovascular mortality compared to matched controls after one, three and five years (7.9 vs. 1.3%; 11.1 vs. 2.4% and 12.3 vs. 3.9%), respectively (42). Surprisingly, the mortality risk was higher in patients without a prior history of hypertension, cardiovascular disease and diuretic medication (HR 7.28, HR 6.59 and HR 5.75). Compared to other autoimmune bullous diseases such as pemphigus, BP shows the highest risk for hypertension, diabetes and cardiovascular disease (36).

**Table 2:** Association of bullous pemphigoid with metabolic, cardiovascular and kidney diseases and their medication.

Author	Year	Location	Study design	Reference cohort	N (case/control)	Metabolic comorbidities and their medication in individuals with BP	OR/*HR*	95% CI	*p*
Marzano A.V., et al. (33)	2012	Italy	Case-control	age-, body mass index- and sex-matched controls	15/28	**Lower 25-OHD-levels**	**n.a.**	**n.a.**	**0.01**
Sarre M.E., et al. (34)	2016	France	Case-control	n.a.	31/59	**Hypovitaminosis D**	**3.7**	**n.a.**	**0.046**
Karabay E. A., et al. (35)	2017	Turkey	Cohort-study	n.a.	62/217	**Patients with BP with higher incidence of** **- Hypertension** **- Diabetes** **- Coronary artery disease**	**n.a.** **n.a.** **n.a.**	**n.a.** **n.a.** **n.a.**	**<0.01** **<0.01** **0.001**
Ren Z., et al. (27)	2017	USA	Register study of hospitalized adult patients in the USA	n.a.	2,108 BP primary diagnosis 11,234 BP secondary diagnosis 72,108,077 total hospital discharges	**Myocarditis** **Diabetes** **Obesity** **Congestive heart failure** **Pulmonary circulation disorders** **Hypertension**	**6.29** **1.57** **1.56** **1.43** **1.38** **0.98**	**1.57-25.1** **1.35-1.82** **1.44-1.70** **1.35-1.52** **1.22-1.58** **0.93-1.02**	**0.02** **0.003** **0.003** **0.003** **0.003** **0.003**
Kwa M. C., et al. (36)	2017	USA	Register study of hospitalized adult patients in the USA	n.a.	13,342/ 72,651,487	**Congestive heart failure** **Pulmonary circulation disorder Hypertension** **Peripheral vascular disease** **Type 2 Diabetes** **Coronary artery disease**	**2.58** **1.92** **1.80** **1.68** **1.45** **1.17**	**2.42-2.74** **1.69-2.19** **1.71-1.90** **1.54-1.83** **1.24-1.69** **1.10-1.24**	**<0.001** **<0.001** **<0.001** **<0.001** **<0.001** **<0.001**
Sim B., et al. (26)	2017	Singapore	Case-control	Age- and sex-matched controls	105/315	**Hypertension** Diabetes Ischemic heart disease Hyperlipidemia Kidney disease	**2.37** 1.23 1.12 0.24 0.44	**1.18-4.77** 0.78-1.96 0.68-1.82 0.1-0.49 0.2-0.99	**0.015** 0.375 0.66 0.001 0.049
Jeon H.W., et al. (7)	2018	Korea	Cohort study	Age- and sex-matched general population of Korea	103/n.a.	**Standardized mortality ratio** **- 70-79 years** **- >80 years** **- total** Prevalence of **Diabetes** Arterial hypertension Chronic kidney disease	***2.35*** ***2.18*** ***1.83*** **1.64** 0.63 0.56	**1.52-3.47** **1.31-3.42** **1.34-2.46** **1.06-2.55** 0.42-0.95 0.30-1.08	**<0.05** **<0.05** **<0.05** **0.026** 0.036 0.109
Varpuluoma O., et al. (37)	2018	Finnland	Register study	Age-, sex and year of diagnosis matched controls	3,397/12,941	Increased risk for BP after adjustment for diabetes and several neurologic disorders **- DPP-4-inhibitors** - Metformin	**2.19** 1.05	**1.55-3.11** 0.88-1.24	**<0.05** >0.05
Varpuluoma O., et al. (38)	2018	Finnland	Register case-control study	Age-, sex and year of diagnosis matched controls	3,397/12,941	- Diabetes prevalence of 19.6% in BP patients - No association of anti-diabetic medication and BP (excluding DPP4-inhibitos, metformin and insulin)	n.a.	n.a.	>0.05
Kalińska-Bienias A., et al. (28)	2019	Poland	Case-control	Age- and sex-matched controls	218/168	**Arterial hypertension**	**2.17**	**1.35-3.49**	**<0.001**
Chovatiya R., et al. (39)	2020	USA	Register study	n.a.	8,864/ 198,102,435	**Osteoporosis** **Pathological fractures**	**1.55** **1.52**	**1.39-1.73** **1.22-1.88**	**<0.0001** **0.0002**
Lee S., et al. (40)	2021	USA	Case-control	Age- and sex-matched controls	91/546	**End-stage renal disease** **Diabetes mellitus** **Chronic kidney disease** **Hypertension**	**3.82** **2.59** **2.29** **2.03**	**2.45-11.1** **1.60-4.19** **1.19-4.40** **1.24-3.32**	**0.0235** **0.0011** **0.0473** **0.0235**
Wu C.Y., et al. (41)	2021	Taiwan	Cohort-study of diabetic patients with and without DPP4-i treatment	Matched by age, sex, duration of diabetes, insulin usage, and propensity score-matching of comorbidities	124,619 DPP4-I/ 124,619 Non-DPP4-I	**Increased risk for BP by** **- Renal disease** **- DPP4-Inhibitor treatment** **- Metformin**	**2.32** **2.15** **1.93**	**n.a.** **1.18-3.91** **n.a.**	**<0.001** **0.01** **0.006**
Sánchez-García V., et al. (2)	2022	Spain	Retrospective observational study	Compared to pemphigus patients	5,424/1,950	Higher prevalence in BP compared to pemphigus **- Arterial hypertension** **- Kidney disease** **- Heart failure** **- Chronic obstructive pulmonary disease** **- Diabetes** **- Osteoporosis**	**n.a.** **n.a.** **n.a.** **n.a.** **n.a.** **n.a.**	**n.a.** **n.a.** **n.a.** **n.a.** **n.a.** **n.a.**	**0.004** **<0.001** **<0.001** **<0.001** **<0.001** **0.003**
Martin E., et al. (13)	2022	Germany	Case-control	Age- and sex-matched controls	300/583	**Renal impairment** **Anemia** **Diabetes mellitus**	**2.21** **2.12** **1.41**	**1.64-2.99** **1.53-2.95** **1.03-1.93**	**<0.0005** **<0.0005** **0.029**
Titou H., et al. (31)	2022	Morocco	Case-control	Age- and sex-matched controls	81/162	No significant association of BP with - Hypertension - Type 2 diabetes - Dyslipidemia	n.a. n.a. n.a.	n.a. n.a. n.a.	0.891 0.062 0.643
Shen W.C., et al. (42)	2022	Taiwan	Cohort study	Age- and sex- matched controls	252/1,008	**Cardiovascular mortality**	***4.8***	3.07-7.59	**<0.05**
Zhang B. et al. (43)	2022	China	Case-control	Age- and sex-matched controls	162/162	**Diabetes** **Obesity** Hypertension	**1.87** **1.80** 0.75	**1.02-3.39** **1.02-3.18** 0.44-1.12	**0.001** **<0.001** 0.200

Data in bold indicate statistically significant results.

BP, bullous pemphigoid; OR, odds ratio; HR, hazard ratio; CI, confidence interval; n.a, not available.

A case-control study including 91 patients and 546 age- and sex-matched controls in the United States showed a significant association of BP with arterial hypertension (OR 2.03; 95% CI 1.24–3.32), diabetes mellitus (OR 2.59; 95% CI 1.60–4.19), chronic kidney disease (OR 2.29; 95% CI 1.19–4.40) and end-stage renal disease (OR 3.82; 95% CI 1.48–9.85) (40). Importantly, anti-diabetic medications, especially dipeptidyl peptidase-4 (DPP-4) inhibitors (HR 2.15; 95% CI 1.18-3.91, p = 0.01) and metformin (HR 1.93; p = 0.006), increase the risk for BP (41) rising the chicken-and-egg-question whether diabetes itself or antidiabetic medication increases the risk for BP. A Finnish registry-based study confirmed that 19.6% of patients with BP suffer from diabetes but subanalyses revealed that oral antidiabetic medication such as sulfonylurea, thiazolidinedione and glucose-like-peptid-1 analogues were not associated with an increased risk for BP (38). Previously, the same study group demonstrated that the use of DPP-4-inhibitors was significantly associated with an increased risk of developing BP (OR 2.19, 95% CI 1.55-3.11), whereas the association with metformin was not significant (OR 1.49; 95% CI 1.32-1.68) (37). These results are in line with a Taiwanese cohort study which observed that the cumulative incidence of BP was significantly higher in a DPP-4-inhibitor-treated cohort than in the non-DPP-4-inhibitor control group over a period of 6 years (0.74 per 1000 vs 0.38 per 1000, p = 0.001) (41). A case-control study from Israel reported a 3-fold increased risk for developing BP in patients treated with DPP-4-inhibitors (OR 3.2; 95% CI 1.9-5.4) (44).

The reported association of BP with chronic kidney disease may be responsible for a strong association of BP with anemia that we recently demonstrated in a case-control study (OR 2.13; CI 1.53–2.95, p < 0.0005) (13). Further analysis revealed that normocytic normochromic anemia, as seen in patients with chronic kidney disease (45), was the predominant form of anemia observed in BP patients. Similar to anemia, osteoporosis is associated with chronic kidney disease (46). A cross-sectional study from the United States that included 8,864 patients with BP showed an increased OR for osteoporosis (1.55; 95% CI 1.39-1.73) and pathological fractures (1.52; 95% CI 1.22-1.88) (39). Patients with long-term systemic steroid treatment were at even higher risk for osteoporosis. Possible pathogenetic links between BP and osteoporosis next to systemic steroid therapy and chronic kidney disease are chronic systemic inflammation with enhanced bone resorption, decreased physical activity and hypovitaminosis D (39). 25-hydroxy-vitamin D (25-OHD), the precursor of vitamin D, is determined in the blood to assess the vitamin D status. In patients with BP lower 25-OHD levels (mean ± standard deviation (SD): 9.6 ± 7.2 ng mL(-1); controls: 22.6 ± 18.7 ng mL(-1), p = 0,022) and a higher prevalence of severe hypovitaminosis D than in controls (73% vs. 27%, p = 0.01) were recorded (33).

### Malignancies

Thirteen of the selected 48 studies focused on malignant diseases in patients with BP (**Table 3**). The association of BP with malignancies has been discussed for a long time. First reports debating a relationship date back to the year 1968. Twelve out of 103 patients with estimated BP subsequently developed malignancy (50). In the following decades several studies addressed this association with contradictory results. In 2021, a Turkish case-control study with 145 BP patients showed higher rates of malignancies compared to controls (OR 2.597; 95% CI 1.328-5.076; p = 0.005). The type of malignancy was not reported but skin cancers were excluded and subgroup analysis found men to be at higher risk than women (OR 4.347; 95% CI 1.488–12.658; p = 0.004) (30).

**Table 3:** Association of bullous pemphigoid and malignancy.

Author	Year	Location	Study design	Reference cohort	N (case/control)	Malignant comorbidities in individuals with BP	OR/*HR*	95% CI	*p*
Lindelöf B., et al. (17)	1990	Sweden	Register study	Age- and sex-matched reference cohort	497	Expected number of incident cancer diagnosis in individuals with BP compared to matched standard incidence ratio showed no significant difference	n.a.	n.a.	n.a.
Ong E., et al. (47)	2011	England	Cohort-study of hospital admission data	reference cohort without known association with either cancer or BP	2,873,729 individuals with cancer 4,720 individuals with BP	Risk of BP in individuals with vs. without cancer **- with lymphoid leukaemia** **- with kidney cancer** **- with laryngeal cancer** Risk of cancer in individuals with BP	0.96 **2.27** **2.23** **2.22** 1.00	0.88-1.04 **1.21-3.89** **1.48-3.24** **1.21-3.75** 0.92-1.09	>0.05 **<0.05** **<0.05** **<0.05** >0.05
Cai S.C.S., et al. (48)	2015	Singapore	Cohort-study	Age- and sex-matched general population	359/n.a.	Expected number of incident cancer diagnosis after BP compared to matched standard incidence ratio	0.97	0.53-1.62	n.a.
Schulze F., et al. (14)	2015	Germany	Cohort-study	Age- and sex-matched controls	1,743/10,141	**Mature T/NK-cell lymphoma Myeloid leukemia** **Hodgkin disease** **Non-follicular lymphoma** **Leukemia of unspec. cell type Unspec. non-Hodgkin lymphoma**	**6.9** **5.7** **4.2** **3.8** **2.7** **2.6**	**3.1-16.0** **1.90-17.0** **1.90-8.9** **1.90-7.5** **1.40-5.1** **1.60-3.9**	**<0.001** **<0.001** **<0.01** **<0.01** **0.03** **<0.01**
Atzmony L., et al. (15)	2017	n.a.	Meta-analysis of cross-sectional, case-control and cohort studies	n.a.	n.a.	**Pooled analysis of cross-sectional studies found significant association of BP and hematologic malignancies but not with overall cancer**	**n.a.**	**n.a.**	**n.a.**
Ren Z., et al. (27)	2017	USA	Register study of hospitalized adult patients in the USA	n.a.	2,108 BP primary diagnosis 11,234 BP secondary diagnosis 72,108,077 total hospital discharges	BP was not associated with higher odds of any solid-organ or hematological malignancies	n.a.	n.a.	>0.1
Kalińska-Bienias A., et al. (28)	2019	Poland	Case-control	Age- and sex-matched controls	218/168	Malignancy	1.74	0.84-3.65	0.13
Kridin K., et al. (16)	2021	Israel	Cohort-study	Age-, sex- and ethnicity-matched controls	3,924/19,280	**Risk of uterine cancer** Risk of solid malignancies History of malignancy	***2.56*** *0.9* 1.00	**1.39-4.72** 0.77-1.05 0.90-1.10	**0.003** n.a. 0.469
Kilic Sayar S., et al. (30)	2021	Turkey	Case-control	Age- and sex-matched controls	145/310	**Preexisting Malignancy**	**2.59**	**1.32-5.07**	**0.005**
Martin E., et al. (13)	2022	Germany	Case-control	Age- and sex-matched controls	300/583	No association between malignancies and BP was observed	n.a.	n.a.	>0.05
Kridin K., et al. (49)	2022	Israel	Case-control	Age-, sex- and ethnicity-matched controls	3,924/19,280	**History of melanoma** Risk of melanoma **Higher prevalence of melanoma in BP patients**	**1.53** 1.13 **n.a.**	**1.14-2.06** 0.73-1.74 **n.a.**	**0.004** 0.587 **0.004**
Shen W.C., et al. (42)	2022	Taiwan	Cohort-study	Age- and sex- matched controls	252/1,008	**Cancer mortality**	***1.87***	**1.01-3.45**	**<0.05**
Titou H., et al. (31)	2022	Morocco	Case-control	Age- and sex-matched controls	81/162	**Significant association of BP with** **Malignancy**	**n.a.**	**n.a.**	**0.017**

Data in bold indicate statistically significant results.

BP, bullous pemphigoid; OR, odds ratio; HR, hazard ratio; CI, confidence interval; n.a, not available.

In contrast, six studies and a meta-analysis observed no significantly increased overall risk for malignancy or cancer-related mortality in patients with BP (13, 15–17, 47, 48, 51). For example, an Asian study including 359 patients with BP reported no increased standard incidence ratio (IR) of malignant diseases compared to sex- and age-matched controls (IR 0.97; 95% CI 0.53-1.62) (48). In line, a British cohort study observed no increased risk for developing BP in patients with a prior history of malignant diseases (risk ratio (RR) 0.96; 95% CI 0.88–1.04) (47). Interestingly, in sub-analyses patients with a history of kidney cancer (RR 2.23; 95% CI 1.48–3.24, p < 0.001), laryngeal cancer (RR 2.22; 95% CI 1.21–3.75; p = 0.004) or lymphoid leukemia (RR 2.27; 95% CI 1.21–3.89, p = 0.005) were at higher risk for developing BP. A German cohort study observed an association of BP with hematological malignancies (OR 2.55; 95% CI 2.07-3.13; p < 0.001) but not with non-hematological malignancies (14). Further analyses found a strong association with Morbus Hodgkin (OR 4.2; 95% CI 1.90–8.9; p < 0.01), non-follicular lymphoma (OR 3.80; 95% CI 1.90–7.5; p < 0.01), mature T/NK-cell lymphomas (OR 6.90; 95% CI 3.1–16.0; p < 0.001), unspecified types of Non-Hodgkin’s lymphoma (OR 2.60; 95% CI 1.60–3.9; p < 0.001), myeloid leukemia (OR 5.70; 95% CI 1.90–17.0; p = 0.01), and leukemia of unspecified cell type (OR 2.70; 95% CI 1.40–5.1; p = 0.03). However, the authors assumed it to be unlikely for the hematological malignancy to have triggered BP as the malignancy preceded BP in only about half of the cases.

A population-based cohort study with an added case-control design found an increased risk of 50% for patients with preexisting melanoma to develop BP (OR 1.53; 95% CI 1.14–2.06) (49). Males (OR 1.66; 95% CI 1.09–2.54) and patients older than 80 years (OR 1.63; 95% CI 1.11–2.38) were at higher risk. Vice versa, the risk for melanoma in patients with BP was elevated but did not reach significance. The increased risk for BP in patients with melanoma persisted even after adjustment for confounders such as therapy by programmed cell death-1/programmed cell death ligand-1 (PD-1/PDL-1) inhibitors.

Immunomodulatory therapy of tumor diseases can also trigger BP. Since the beginning of immune checkpoint inhibitor therapy, many cases and case-series with PD-1/PDL-1 inhibitor-induced BP have been published (52). Unlike immune checkpoint inhibitor-induced maculopapular rash, first symptoms of BP, e.g. pruritus, often occur months after the start of immunotherapy or even after discontinuation (53). In the majority of published cases development of BP led to a stop of immunotherapy (54).

### Autoimmune and inflammatory diseases

Eleven of 48 studies discussed autoimmune and inflammatory diseases in patients with BP (**Table 4**). The association of BP with MS is well established as described above. In 1981, a case report of concomitant BP and rheumatoid arthritis initiated an ongoing discussion about the association of BP and autoimmune or inflammatory diseases (64). A case-control study published in 1993 focused on the association between autoimmune disorders such as diabetes mellitus type 1, rheumatoid arthritis, alopecia areata and BP. No differences were detected between BP patients and age- and sex-matched controls (55).

**Table 4:** Association of bullous pemphigoid with autoimmune and inflammatory diseases.

Author	Year	Location	Study design	Reference cohort	N (case/control)	Autoimmune and autoinflammatory comorbidities in individuals with BP	OR	95% CI	*p*
Taylor G., et al. (55)	1993	United Kingdom	Case-control	Age- and sex-matched controls	108/108	- Incidence of autoimmune disorders between patients and controls was not significant - The haplotypes of patients with BP were similar to those of a locally drawn population	n.a.	n.a.	n.a.
Ameri P., et al. (56)	2013	Italy	Retrospective cohort-study	n.a.	23/46	**- Thyroid autoantibody positivity** **- anti-TPO autoantibody positivity**	**6.75** **6.27**	**1.55-31.5** **1.23-35.5**	**<0.01** **<0.05**
Kridin K., et al. (57)	2017	Israel	Case-control study	Age-and sex- and ethnicity-matched controls	287/1,373	**Psoriasis**	**4.4**	**2.2-8.9**	**<0.0001**
Phan K., et al. (58)	2019	Australia	Meta-analysis of case-control studies	n.a.	4,035/19,215	**Psoriasis**	**2.5**	**1.4-4.6**	**0.003**
Kridin K., et al. (21)	2020	Israel	Retrospective population-based cohort-study and case-control study	Age-, sex- and ethnicity-matched controls	3,924/19,280	**Psoriasis in individuals with BP** **BP in individuals with psoriasis**	**2.60** **1.53**	**1.59-4.27** **1.17-2.02**	**<0.001** **0.002**
Chen Y.J., et al. (59)	2020	Taiwan	Case-control study using a nationwide database	age-, gender- and hospital visit number-matched controls	5,263/21,052	**Ulcerative colitis**	**3.60**	**1.91-6.77**	**<0.001**
Kridin K., et al. (60)	2021	Israel	Population-based -study	Age-, sex- and ethnicity-matched controls	3,924/19,280	**History of Asthma**	**1.43**	**1.30-162**	**<0.001**
Ständer S., et al. (61)	2021	Germany	Retrospective cohort study	Comparison of BP patients with and without psoriasis	274 (11/262)	Prevalence of psoriasis 4% **- younger age at onset of BP** **- milder erosive phenotype** **- lower levels of BP-180 antibodies**	n.a.	n.a.	n.a. **0.023** **0.025** **0.008**
Kridin K., et al. (62)	2022	Israel	Population-based cohort-study and case-control study	Age-, sex- and ethnicity-matched controls	3,924/19,280	**History of atopic dermatitis (AD)** **History of allergic rhinitis (AR)** **Risk of AD** Risk of subsequent AR	**1.76** **1.13** **2.00** 1.00	**1.44-2.15** **1.01-1.28** **1.60-2.51** 0.83-1.20	**<0.001** **0.047** **<0.001** 0.997
Wu P.C., et al. (19)	2022	Taiwan	Two case-control studies using a nationwide database	1) age- and sex-matched controls 2) age-, sex-, and propensity score of comorbidities matched controls	1) 9,344/18,688 2) 7,196/14,392	**1)** **Psoriasis** **Atopic dermatitis** **2)** **Atopic dermatitis**	**2.83** **1.71** **1.76**	**2.27-3.51** **1.50-1.95** **1.55-2.00**	**<0.001** **<0.001** **<0.001**
Kridin K., et al. (63)	2022	Israel	Population-based cross-sectional study	Age- and sex-matched controls	1,743/10,141	**Thyreoiditis**	**1.98**	**1.18-3.35**	**0.010**

Data in bold indicate statistically significant results.

BP, bullous pemphigoid; OR, odds ratio; HR, hazard ratio; CI, confidence interval; n.a, not available.

In the last few years several studies investigating the association of psoriasis and BP have been published. A meta-analysis of four case-controls studies including 4,035 BP patients and 19,215 controls revealed a significantly higher prevalence of psoriasis in patients with BP than in controls (2.6% vs. 1.1%; OR 2.5; 95% CI 1.4-4.6), predominantly in males compared to females (3.0% vs. 1.9%; OR 1.75; 95% CI 1.1-2.7) (58). A large population-based retrospective cohort study including 3,924 patients with BP with age-, sex- and ethnicity-matched controls reported a higher prevalence of preexisting psoriasis in patients with BP than in controls (1.7 vs. 1.1%, p < 0.001) (21). Additionally, patients with a history of psoriasis had a higher risk of developing BP (OR 1.53; 95% CI 1.17–2.02) and patients with BP had a 2.6-fold increased risk of developing psoriasis as compared to controls (HR 2.60; 95% CI 1.59–4.27).

Next to psoriasis, patients with a preexisting diagnosis of atopic dermatitis or allergic rhinitis show an increased risk for subsequent BP (OR 1.76; 95% CI 1.44-2.15; p < 0.001 and OR 1.13; 95% CI 1.01-1.28; p = 0.047) (62). These findings are in line with a recent large population-based case-control study that identified atopic dermatitis to be a significant risk factor for BP compared to age-, sex-, and comorbidity-matched controls (OR 1,76, 95% CI 1,55–2,00) (19). Next to atopic dermatitis and allergic rhinitis asthma is associated with a 50% increased risk of developing BP (OR 1.45; 95% CI 1.30–1.62) and the prevalence of preexisting asthma was higher in patients with BP than in controls (11.1 vs. 7.9%; p < 0.001) (60).

Inflammatory bowel disease is characterized by a non-infectious inflammation mediated by inflammatory cytokines that lead to mucosal destruction. Skin diseases such as erythema nodosum and pyoderma gangraenosum are well-known to occur more frequently in patients with inflammatory bowel disease. Only few data regarding the association of Inflammatory bowel disease and BP have been published. In 2020, a population-based case-control study reported a significant association of BP with ulcerative colitis (OR 3.60, 95% CI 1.91–6.77, p < 0.001) but not Crohn’s disease (59).

Inflammatory diseases of the thyroid including Graves’ disease and Hashimoto thyroiditis are the most common autoimmune disorders with a prevalence of 2-5% predominantly affecting women (65). Several case reports of patients with autoimmune thyroid diseases who later developed BP have been published suggesting an association of these autoimmune disorders (66, 67). Recently, a significant association between thyroiditis and BP (OR 1.98; 95% CI 1.18-3.35; p = 0.01) has been reported (63). An Italian hospital-based study observed significantly higher anti-TPO-autoantibody levels in patients with BP but not in patients with pemphigus vulgaris compared to controls (56).

## Discussion

### Neurologic and psychiatric diseases

The association of BP with neurologic and psychiatric diseases is well acknowledged and confirmed by several case-control and cohort studies (8–13). BP-180, the main autoantigen in BP, is also expressed in the central nervous system (68). A prospective multicenter study including 499 patients with BP found an association between neuropsychiatric disease and increased BP-180 autoantibody seropositivity (69). Likewise in sera of 115 patients with Alzheimer’s disease levels of BP-180 autoantibodies were significantly increased compared to controls, however, these antibodies failed to react with cutaneous basement membrane in indirect immunofluorescence analysis and none of the patients had clinical symptoms of BP (70). Next to BP-180, BP-230 is another major autoantigen in BP. Several isoforms of BP-230 are existing; among others the epithelial isoform BP-230-e connects hemidesmosomes to keratin filaments. BP-230-a is detected in the nervous system, maintaining the organization of the microtubule network in neurons. Possibly, patients with MS can develop an autoantibody response to neuronal variants of BP-230 (71). Due to the degeneration of nerves and their sheaths, structural proteins such as BP-230 may be recognized by the immune system and trigger autoinflammation. This may result in an autoimmune response against the epithelial isoform of BP-230 and development of BP (72). In mouse models sera of patients with both BP and neurologic diseases reacted with BP-230 proteins from mouse brain extract (73). In line with these results, a single-center retrospective case–control study from Turkey observed significantly higher BP-230 and BP-180 autoantibody titers at initial diagnosis of BP in patients with preexisting neurological disorders than without (30). Similar results were observed in a German retrospective study that reported a higher BP-230 autoantibody seropositivity in BP patients with comorbid neuropsychiatric disease than in those without (67.7% vs. 36.5%; P = 0.006); moreover, coexistence of BP and neuropsychiatric disease was significantly associated with anti-BP-230 seropositivity (OR 3.43; 95% CI 1.24-9.52; p = 0.018) (74). This possible pathomechanism suggests that the degeneration of nerves fosters the development of autoantibodies that cross-react with the skin. However, in the follow-up phase of the Danish registry-based cohort study mentioned above (10), the HR for the development of MS was significantly higher in patients with BP than in control subjects, raising the question of whether there are other, yet unknown mechanisms linking the two diseases.

### Cardiovascular and metabolic diseases

Similar to BP, inflammation is the major trigger of the early phases of the atherosclerotic process and inflammatory cytokines are associated with a higher risk for developing cardiovascular diseases (75). Next to BP-230-a and BP-230-e, a third isoform named BP-230-b is known (76). This isoform is mainly expressed in the myocardium and skeletal muscle of mice (77). It seems likely that, similar to neurologic diseases, cross-reactivity of autoantibodies to exposed structural proteins may promote the development of BP, but evidence is still lacking. On the contrary, a prospective multicenter study including 499 patients with BP found no association of elevated BP-180 autoantibody serum levels with metabolic disorders (including diabetes mellitus type 2) and arterial hypertension (69). Interestingly, medication with DPP-4-inhibitors in patients with BP was associated with lower BP-180 and BP-230 autoantibody seropositivity compared to BP patients without DPP-4-inhibitor intake (69). Several studies reported clinical and serologic differences in DPP-4 inhibitor-induced BP compared to classic BP, such as they were more likely to show a noninflammatory phenotype, lower eosinophil counts and lower autoantibody seropositivity (78, 79). Although the exact mechanisms of induction of BP by DPP-4-inhibitors have not been finally elucidated yet, these drugs should only be used with caution in BP patients especially if alternative therapies are available. As patients with BP seem to be at higher risk for osteoporosis, pathological fractures and hypovitaminosis D they should be encouraged to be physically active and to substitute vitamin D if necessary.

Altogether, study data are inconclusive with respect to associations between BP and metabolic diseases as well as chronic kidney disease and arterial hypertension; however, since we are facing an older patient population, these diseases should certainly be considered.

### Malignancies

The association of BP and malignancy is not conclusively proven or disproven. Interpretation of the data remains challenging since multiple confounders such as ethnicity (including variability in HLA genotypes (80, 81)), age, gender and lifestyle must be considered regarding the association of malignancy and BP. New data suggests associations of BP and melanoma (49); however, it should be kept in mind that both patients with BP and melanoma are usually examined by dermatologists, thus, the latter may be detected earlier and more frequently.

As BP affects an elderly population, malignancy is common but might not be necessarily associated with the skin disease by itself.

### Autoimmune and inflammatory diseases

An ongoing discussion about the association of BP and autoimmune or inflammatory diseases already started in 1981 (64). In recent years new evidence reignited the discussion when inflammatory skin diseases such as psoriasis and atopic dermatitis were reported to be associated with BP.

Interestingly, patients with BP and comorbid psoriasis were significantly younger, presented with a milder erosive phenotype and lower levels of BP-180 autoantibodies (61). This leads to the question of how BP and psoriasis influence each other. Multiple hypotheses have been postulated addressing this question; it has been suggested that (i) impairment of the basal membrane in psoriasis may lower the threshold for the generation of anti-basal membrane autoantibodies, (ii) shared pathogenetic features such as inflammation mediated by neutrophils, interleukin-1 (IL-1) and -17 (IL-17) and (iii) anti-psoriatic medication may trigger BP. Degradation of proteins in the basal membrane such as laminin 1 in psoriasis can modify the antigenicity of the basal membrane and therefore possibly enhance the formation of autoantibodies that are found in BP (82). Neutrophils are present in lesional skin of both diseases and may promote destruction of matrix proteins of the basal membrane *via* secretion of metalloproteinases exposing antigens of the dermal-epidermal junction and thus contributing to the generation of autoantibodies (83). IL-1, IL-17 and T-helper 17 (Th-17) cells play a major role in the pathogenesis of psoriasis; interestingly, IL-1 beta was found in blister fluid of BP (84) and Th-17 cells were significantly increased in conjunctival biopsies of patients with ocular cicatricial pemphigoid (85). Whether these findings are causal or merely coincidental must be clarified by further investigations. On the other hand, treatment of psoriasis may trigger BP as several case reports described development of BP after treatment with PUVA or biologicals such as adalimumab, efalizumab, etanercept, ustekinumab and secukinumab, respectively (86, 87).

BP also shares pathogenetic aspects with atopic dermatitis, allergic rhinitis and asthma such as elevated serum levels of immunoglobulin E (IgE), eosinophilia and a Th-2 response (88, 89). Additionally, dupilumab, a monoclonal antibody inhibiting Th-2 cytokines IL-4 and IL-13, has been successfully applied for treatment of BP (90).

## Conclusion

BP is frequently accompanied by comorbid diseases. For some conditions, e.g. neuropsychiatric disorders, a clear association with BP has consistently been demonstrated in several studies suggesting that they share common pathomechanistic attributes. For other diseases the data situation is ambiguous and further, ideally prospective investigations are necessary to more reliable estimate an association with BP.

Several observations point to a pathomechanistically based association between BP and other diseases and conditions. These include: (i) Formation of autoantibodies due to augmented exposure to human structural proteins following cell damage (due to degenerative diseases, malignancies, inflammation); (ii) underlying common canonical inflammatory pathways of BP and other autoimmune and inflammatory diseases; (III) exposure to medication that subsequently facilitates formation of autoantibodies.

In our opinion, the results of the available studies do not currently allow a general screening recommendation for comorbid diseases in BP beyond a thorough work-up including but not limited to a detailed medical history, surveying comedication and vital signs, medical examination and routine laboratory testing. Hereby, most of the commonly associated comorbidities should be detected. In case of doubt, referral to a specialist such as a neurologist should be considered. Treating physicians should be aware of commonly associated diseases such as neurological, cardiovascular, malignant and inflammatory disorders in order to treat the patient in the best possible way and to avoid therapy-associated complications.

## Data availability statement

The original contributions presented in the study are included in the article/supplementary material. Further inquiries can be directed to the corresponding author.

## Author contributions

JH conducted literature searches, drafted the manuscript and finalized the manuscript for submission. SB and MG edited drafts of the manuscript and finalized the manuscript for submission. MG initiated the review. All authors contributed to the article and approved the submitted version.
